# Role of the Primary Motor Cortex in the Early Boost in Performance Following Mental Imagery Training

**DOI:** 10.1371/journal.pone.0026717

**Published:** 2011-10-26

**Authors:** Ursula Debarnot, Emeline Clerget, Etienne Olivier

**Affiliations:** Institute of Neuroscience, Université catholique de Louvain, Brussels, Belgium; Harvard Medical School, United States of America

## Abstract

Recently, it has been suggested that the primary motor cortex (M1) plays a critical role in implementing the fast and transient post-training phase of motor skill consolidation, known to yield an early boost in performance. Whether a comparable early boost in performance occurs following motor imagery (MIM) training is still unknown. To address this issue, two groups of subjects learned a finger tapping sequence either by MIM or physical practice (PP). In both groups, performance increased significantly in the post-training phase when compared with the pre-training phase and further increased after a 30 min resting period, indicating that both MIM and PP trainings were equally efficient and induced an early boost in motor performance. This conclusion was corroborated by the results of an additional control group. In a second experiment, we then investigated the causal role of M1 in implementing the early boost process resulting from MIM training. To do so, we inhibited M1 by applying a continuous theta-burst stimulation (cTBS) in healthy volunteers just after they learnt, by MIM, the same finger-tapping task as in Experiment #1. As a control, cTBS was applied over the vertex of subjects who underwent the same experiment. We found that cTBS applied over M1 selectively abolished the early boost process subsequent to MIM training. Altogether, the present study provides evidence that MIM practice induces an early boost in performance and demonstrates that M1 is causally involved in this process. These findings further divulge some degree of behavioral and neuronal similitude between MIM and PP.

## Introduction

Skill acquisition is characterized by at least two distinct phases: a fast, within-session learning stage followed by a delayed and time-dependent one [1]–[3]. This late phase is regarded as the outcome of a memory consolidation process, leading to the transformation of a given experience into a stable and long-lasting form [4]. A distinctive stage of motor memory consolidation is an early transient increase in performance known as the “early boost”, which occurs after a short period of time following training, typically 5–30 minutes [5]–[10]. Recently, Hotermans et al. [11] investigated the role of the primary motor cortex (M1) in the different phases of motor memory consolidation by applying repetitive transcranial magnetic stimulation (rTMS) over M1 just after a training session involving a sequential finger-tapping task. These authors showed that interfering with M1 attenuated the early boost amplitude, suggesting that M1 is causally involved in this early post-training phase. Their conclusion was that, beyond the classical implication of M1 in motor skill acquisition, this area is also reactivated during the subsequent early boost process. This assumption is consistent with the view that the brain network activated during physical practice (PP) is reactivated at rest [12], a “replay” in neuronal activity which may modify the synaptic connections in the networks activated during PP, strengthening some synaptic connections while weakening others, in order to refine the memory process [13]–[17].

In the wealth of the motor learning literature, mental practice, and most specifically motor imagery (MIM), is regarded as an effective complement to, or even as a substitute for, PP to enhance cognitive and motor performance [18]. MIM is the mental representation of an overt action without performing the actual movement [19], [20]. There is now ample evidence that MIM and PP share several characteristics, at a temporal, behavioral and neural level [21]–[23]. Indeed, many experiments have indicated that the time course of mentally simulated actions is tightly correlated with that required to execute the same movement. Secondly, it has been shown that the autonomic nervous system has a comparable activity during both imagined and actual movements. Finally, functional brain imaging studies have evidenced that both executed and imagined goal-directed movements recruit overlapping - though not strictly identical - neural structures. For example, it has been observed that M1 is activated during MIM, but to a lesser extent than during movement execution [24]–[26]. So far, many investigations have suggested that MIM is effective for learning motor sequences [18], [27] and generates changes in brain activation during and after mental training [28]. Although the effect of MIM practice on motor memory consolidation has been much less investigated than that of PP training, Debarnot and collaborators [29]–[31] have demonstrated that MIM practice yields the same long-term motor memory consolidation process as PP. However, whether an early boost in performance occurs after MIM training and whether M1 contributes to this process are still largely unknown.

The aim of the present study was twofold. First, we tested whether MIM training of a motor sequence task can elicit an early boost in performance, which is comparable to that observed after PP of the same task. Secondly, we investigated whether M1 contributes to this early boost process. To address this particular issue, we applied a continuous theta-burst stimulation (cTBS) over the hand representation of M1 in order to inhibit transiently this brain region and we investigated whether it affected the early boost process; cTBS was delivered just after MIM training on the same motor sequence as that used in Experiment #1. Transcranial magnetic stimulation, and rTMS in particular, has proven to be an efficient non-invasive technique to determine the causal role of a given cortical area in motor or cognitive functions since it allows us to determine the behavioral consequences of a transient disruption of the area under investigation on the task at hand [32], [33]. More recently, a novel TMS protocol, the cTBS, has been introduced [34]. Although it has been shown that cTBS yield an inhibition of an amplitude comparable to that induced by rTMS [35], this technique has two main advantages: 1) TMS application is very short, typically 40 s, - and is therefore less unpleasant for the subjects - whereas its effect persists for about 30 min [35], [36]; 2) TMS is applied off-line, before the subject performs the task, minimizing the possible interference with the task execution. Recent studies have demonstrated the usefulness of this approach in deciphering some cognitive [37] and learning processes [38].

## MethodS

### Experiment #1

The first experiment aimed to investigate whether MIM training can induce an early boost in performance, as already reported in the literature for PP.

#### Participants

Twenty-four healthy volunteers (13 women, age range: 24–37, mean: 28 years) participated in this experiment; they were evenly distributed into three groups (MIM, PP and control, see below). All subjects were right-handed, as assessed by the Edinburgh Handedness Inventory [39]. None of them reported a prior history of drug or alcohol abuse, neurological, psychiatric, or sleep disorders. Additionally, they were asked to be drug, alcohol, and caffeine free for 24 h prior, and during the experiment. Musicians and professional typists were excluded to avoid incorporating subjects with a high experience in finger tapping tasks. This study was approved by the Ethics Committee of the Université catholique de Louvain, and all participants signed a written informed consent form. The experimental procedure was explained and the subjects received instructions about the task, but no information was provided about the objectives of this study.

Before the experiment, all participants were first asked to fill in the Stanford Sleepiness Score (SSS, [40]) questionnaire, which provides a subjective measure of alertness. The SSS is a 7-point scale, with 1 being the most alert state. This questionnaire was presented twice during the experiment, before the pre-training session and then just before the re-test (see below). The mean score to the SSS before the pre-training session was 2.0±0.27 (mean± SD, n = 8) for the MIM group, 1.70±0.18 (n = 8) for the PP group and 1.40±0.18 (n = 8) for the control (Ctrl) group; this mean score was, respectively, 1.88±0.23, 1.75±0.16 and 1.40±0.18 before the re-test. A repeated measures analysis of variance (ANOVA_RM_) with group (MIM, PP *vs.* Ctrl) as between-subjects factor and session (pre-training *vs.* re-test) as within-subjects factor did not show a main effect of group (F_2,21_ = 2.39, P = 0.11) or a group×session interaction (F_2,21_ = 0.45, P = 0.63) on this score, suggesting that the alertness of subjects from all three groups was identical during the whole experiment.

Before the experiment began, subjects from the MIM and PP groups were also asked to fill in the revised version of the Movement Imagery Questionnaire (MIQ-R, [41]), used to assess the subject's ability to form 1) kinaesthetic images and 2) visual mental images. The MIQ-R consists of an 8-item self-report questionnaire, in which participants rate the vividness of their mental representation on a 7-point scale (1 = very hard to see/feel and 7 = very easy to see/feel). For the visual imagery, the MIQ-R scores were 24.20±1.07 in the MIM group and 23.00±0.95 in the PP group; the kinaesthetic imagery scores were 19.60±2.19 and 16.0±1.50, respectively, for the MIM and PP groups. An ANOVA_RM_ with group (MIM *vs.* PP) as between-subjects factor and imagery type (visual *vs.* kinaesthetic) as within-subjects factor showed no main effect of group (F_1,14_ = 1.86, P = 0.20) and no group×imagery type interaction (F_1,14_ = 0.42, P = 0.52), indicating an equivalence between the two experimental groups in terms of ability to elicit mental images.

#### Task

The participants seated on a chair at a distance of about 50 cm in front of a 17-inch computer screen; they were in a quiet room, without any distracting stimuli in order to help them to focus on the task. A computerized version of the sequential finger-tapping task developed by Karni et al. [42] was used to investigate motor sequence learning. The task consisted of performing a five-move sequence using fingers 2–5 of the left hand, each finger being associated to a key on a computer keyboard. The subjects were asked to use their left, non-dominant, hand to minimize inter-individual difference in tapping expertise and to have more homogeneous groups. The subjects had to repeat the sequence (2-5-3-4-2) as quickly and as accurately as possible for a period of 30 seconds. All practice sessions were organized according to a block design alternating 30 seconds of task performance indicated by a black screen followed with 20 seconds of rest indicated by a white screen. At the end of each sequence, they were asked to press the space bar with their right hand in order to allow us to measure the duration of each sequence; the onset of the next sequence was determined when the first key of the sequence was pressed. The timing of each key-press was recorded by means of a homemade MATLAB program (The Mathworks, Inc., Natick, MA, USA) and was used to calculate the mean duration of each sequence. This program also allowed us to detect errors by comparing the sequences actually performed by the participants with the correct one.

#### Experimental procedure

**Figure 1:**
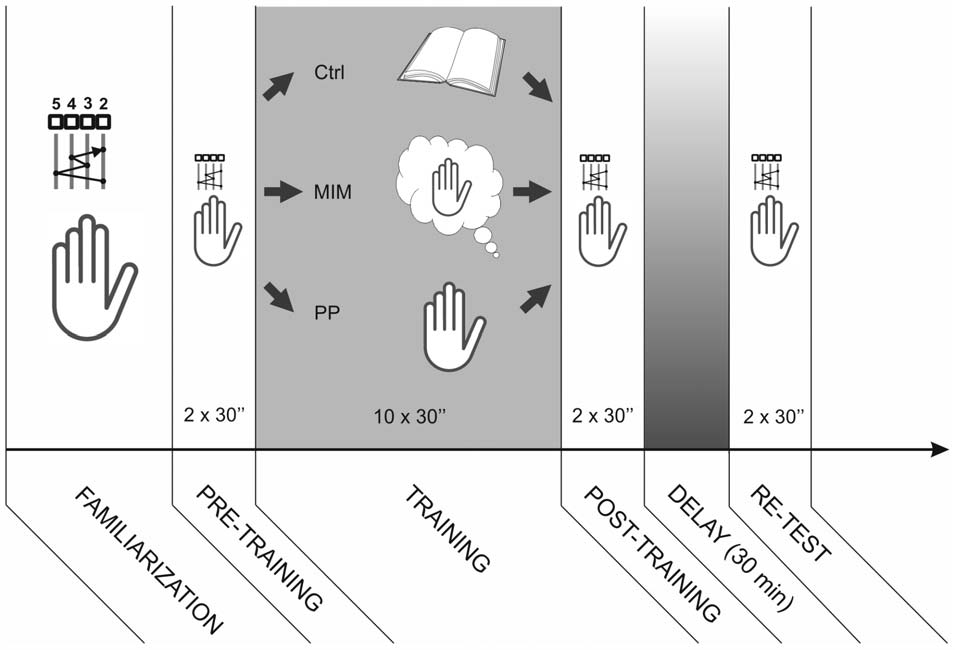
Schematic view of Experiment #1 protocol. The task consisted of performing a sequential finger tapping with the left hand on a computer keyboard. The experiment was divided into six distinct phases: 1) a familiarization phase, 2) a first pre-training, practice session to evaluate subject performance baseline before learning, 3) a training phase during which 3 different group of subjects (n = 8) either physically (PP) or mentally (MIM) performed the sequence (PP) during 10 blocks of 30 seconds, each separated by a 20 second resting period or were engaged in a control (Ctrl) reading task, 4) a first post-training session, 5) a 30 minute delay period, 6) a retest session in order to evaluate the “early boost”.

The experiment was divided into six phases illustrated in Figure 1.

*1)* **Familiarization session.** Buring this session, subjects from all three groups were asked to observe one of the experimenters performing the task. They were then allowed to perform a few trials until they executed five consecutive correct sequences.

*2)* **Pre-training session.** It consisted of two practice blocks lasting 30 seconds each, during which the subjects had to execute the correct sequence as many times as possible. No feedback was provided to the subject about his/her performance. These two practice blocks were separated by a 20 second rest period during which the participants were asked not to imagine or to perform any finger movements.

*3)* **Training session.** Subjects from the PP and MIM groups were asked, respectively, to perform the task physically or to imagine the finger sequence during 10 blocks of 30 seconds each, separated by a resting period of 20 seconds. At the beginning of the experiment, a validated imagery script was read to the MIM participants and briefly repeated right before the MIM training, in order to ensure that they followed the instructions throughout MIM sessions [29]. Subjects from the MIM group were asked to imagine themselves performing the motor sequence using a combination of visual imagery and kinaesthetic imagery, i.e. imagining movement from within one's body and perceiving the sensations induced by executing the sequence. They were also asked to conform to the correct sequence and to imagine its execution at the same speed as during the pre-training session. To prevent any actual finger movements during MIM, the participants were asked to leave their left hand relaxed on their right forearm, and to keep their eyes open in order to see the changes in screen background, indicating the training and rest periods. To be able to record the duration of each finger sequence, participants from both groups were asked to press the space bar with their right hand at the end of each, either executed or imagined, sequence.

During this session, subjects from the control group did not receive any training but, instead, they were asked to read a magazine of their choice [43] during a period of time equivalent to the duration of the training session for the MIM and PP groups, namely 8 minutes. When required, the experimenter turned the pages of the magazine. The control subjects were also asked to remain relaxed and immobile; electromyographic (EMG) activity was recorded from the first dorsal interosseous (FDI) muscle of the left hand, by means of surface electrodes. The EMG signal was continuously monitored and stored on a personal computer; when some EMG activity was noticed in the background, the participants were asked to relax.

*4)* **Post-training session.** This session consisted of two blocks of trials performed by the subjects from all three groups in order to evaluate their performance after the training session (MIM or PP) or the control task. The procedure was the same as that used during the pre-training session: the participants were asked to execute physically the finger sequence as fast and as accurately as possible during two 30 second periods, interleaved with a 20 second resting period. For subjects from the MIM group, individual debriefings were performed to ensure they fulfilled the MIM instructions, and to determine whether they encountered difficulty in forming mental images. Simultaneously, MIM group participants were asked to auto-evaluate the quality of their mental images using a Likert-type scale (from 1 = poor mental representation to 6 = vivid mental representation).

*5)* **Delay.** This first post-training session was followed by a 30 minute delay period. All participants were clearly instructed not to perform any MIM or PP of the sequential finger-tapping task during this delay period.

*6)* **Re-test session.** After this delay, subjects performed a last two-block session in order to evaluate the early boost in performance known to occur after PP training [11]. This re-test session followed the same protocol as that used in the pre-training and post-training sessions.

#### Data analysis

For each practice session (pre-training, post-training and re-test), we analyzed two dependent variables, namely the number of correct sequences (total number in the two blocks) and mean sequence duration. For the MIM group, we also analyzed the duration of each imagined sequence during the training in order to check whether the participants complied with the instructions they received about mental imagery (see above); to do so, we used a Student paired *t*-test to compare the sequence duration in the pre-training and MIM training sessions.

To make sure the initial performance of all three groups was identical, we performed a one-way ANOVA on the number of correct sequences and mean sequence duration gathered in the pre-training test. To investigate the effect of training and the presence of an early boost in the re-test session, we performed an ANOVA_RM_ with group (MIM, PP *vs.* Ctrl) as between-subjects factor and session (pre-training, post-training and re-test) as within-subjects factor. When appropriate, Tukey post-hoc comparisons were performed.

In a subsequent analysis, in order to minimize possible baseline differences between groups, the relative changes in performance (sequence number and duration) in the post-training and re-test sessions were computed and expressed in percent with respect to the pre-training values. To examine the effects of training in the post-training and re-test sessions, we performed an ANOVA_RM_ on these relative values with group (MIM *vs.* PP) as between-subjects factor and session (post-training *vs.* re-test) as within-subjects factor.

Finally to compare the amplitude of the early boost across groups, the change in performance in the re-test session was expressed in percent with respect to the post-training values; then, we performed a one-way ANOVA with group (MIM *vs*. PP) as between-subjects factor.

We used Statistica workpackage (Statsoft Inc., Tulsa, OK, USA) for data analysis. Through this paper, the results are given as mean ± SD, and threshold for significance was set at p<0.05.

### Experiment #2

This second experiment was designed to test the causal role of M1 in the early boost in performance resulting from MIM practice (see Results). To address this issue, we inhibited the right contralateral M1 by using cTBS (M1 group). As a control, cTBS was applied over the vertex in another group of subjects (Vertex group). Because Experiments #1 and #2 used the same task and experimental procedure, only the differences between the two experiments will be highlighted in the following sections.

#### Participants

Sixteen additional volunteers (5 women, age range: 19–37, mean: 25 years) participated in this second experiment. Each subject was seen by a neurologist to rule out any potential risk of adverse reactions to TMS, based on the TMS Adult Safety Screen [44]. This experiment was approved by the Ethics Committee of the Université catholique de Louvain. All participants signed a written informed consent and were compensated for their participation.

#### Task and experimental procedure

The SSS performed during the pre-training session (see above) gave a score of 1.63±0.52 (mean ± SD, n = 8) for the M1 group and of 1.53±0.74 for the Vertex group; it was, respectively, 1.50±0.53 and 1.63±0.52 before the re-test. A one-way ANOVA on these scores showed no main effect of group (F_1,8_ = 1.14, p = 0.06) or group×session interactions (F_1,8_ = 1.17, p = 0.67). The MIQ-R scores were 24.50±1.07 for the M1 group and 22.75±1.51 for the Vertex group for the visual imagery; for the kinaesthetic imagery, the scores were 20.67±1.35 and 19.75±2.24, respectively. An ANOVA_RM_ showed no main effect of group (F_1,8_ = 1.40, p = 0.27) or interaction between group and imagery type (F_1,8_ = 0.77, p = 0.4). The results of these two questionnaires indicated that the two groups were identical in terms of alertness and ability to elicit motor mental images.

**Figure 2:**
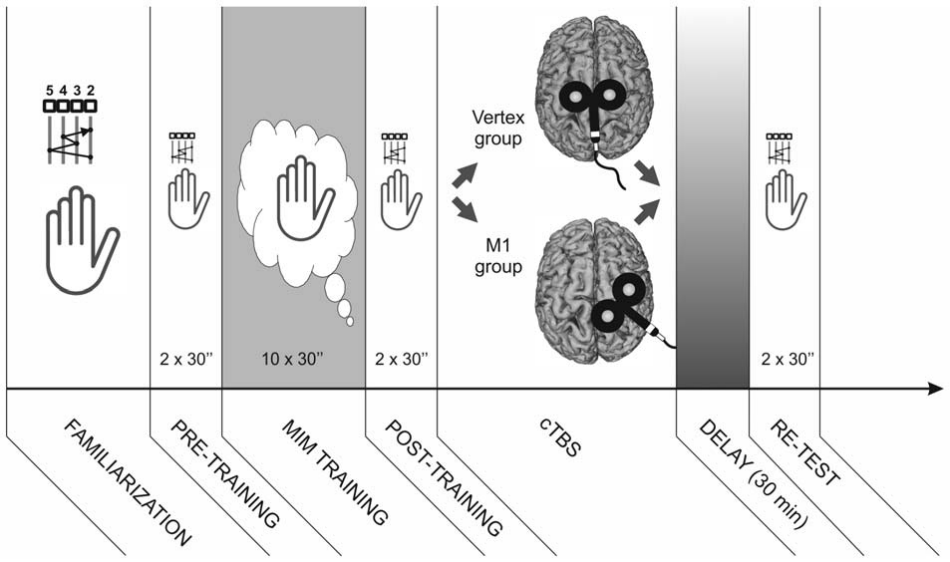
Schematic view of Experiment #2 protocol. The experiment was the same as that undergone by subjects from the MIM group in Experiment #1 except that, after the post-training session, cTBS was applied either over the contralateral (right) primary motor cortex (M1) in a group of 8 subjects or over the vertex in another group of 8 controls subjects.

All participants underwent the same experimental procedure as that already described for Experiment #1 (i.e. familiarization, pre-training, training, post-training, delay and re-test), but both groups of participants underwent a MIM training (Fig. 2). To check that the subjects remained relaxed during MIM training, EMG activity from the FDI was continuously monitored throughout the training phase; if any EMG activity was visible, the participants were asked to relax. As in Experiment #1, during the three test sessions (pre-training, post-training and re-test), the subjects were required to repeat the sequence as quickly and as accurately as possible for two periods of 30 seconds, separated by a 20 second resting period.

#### Transcranial magnetic stimulation

Participants were comfortably seated in an armchair with the left arm relaxed. cTBS was delivered with a Super Rapid stimulator (Magstim Company Ltd, Whitland, UK) through a figure-of-eight coil (external wing 7 cm in diameter). The coil was placed tangentially over the right hemisphere, with the handle pointing backward and away from the midline at about 45°, at the optimal scalp position (hot spot) for eliciting motor evoked potentials (MEPs) in the contralateral, left, FDI muscle. Then, the resting motor threshold (rMT) was determined; rMT is defined as the lowest intensity able to evoke MEPs of at least 50 µV in five out of ten consecutive stimulations. cTBS was applied following the protocol introduced by Huang and collaborators [34]: bursts of three pulses delivered at 50 Hz (80% of rMT) were repeated every 200 ms for 40 s (600 pulses).

#### Data analysis

As in Experiment #1, we analyzed the two following dependent variables: the total number of correct sequences performed in the two blocks of each practice session (pre-training, post-training and re-test) and the mean duration of these sequences. To analyze whether the participants complied with the imagery guidelines, we performed a Student paired *t*-test to compare the mean sequence duration in the pre-training and MIM training session. Then, the relative changes in performance (sequence number and duration) in the post-training and re-test sessions were expressed in percent with respect to the pre-training values and an ANOVA_RM_ was performed on these relative values with group (M1 *vs.* Vertex) as between-subjects factor and session (post-training vs. re-test) as within-subjects factor. The early boost amplitude was measured as the change in performance in the re-test session when compared with the post-training values, expressed in percent; these values across groups were compared by means of a one-way ANOVA with group (M1 *vs.* Vertex) as between-subjects factor.

## Results

### Experiment #1: Evidence for an early boost in performance following MIM training

First, we aimed to determine whether the three groups (MIM, PP and Ctrl) were comparable in terms of performance during the pre-training session. In this session, subjects from the MIM, PP and Ctrl groups performed, respectively, 27.38±2.10, 32.88±3.93 and 30.00±2.22 correct sequences (total number of correct sequences in 2 blocks ± SD, n = 8); their mean sequence duration was 1.72±0.12 s, 1.39±0.16 s and 1.53±0.10 s (mean ± SD, n = 8), respectively. Two separate one-way ANOVA on the number of correct sequences (F_2,21_ = 0.92, p = 0.41) and on the mean sequence duration (F_2,21_ = 1.65, p = 0.21) showed no difference between groups.

**Figure 3:**
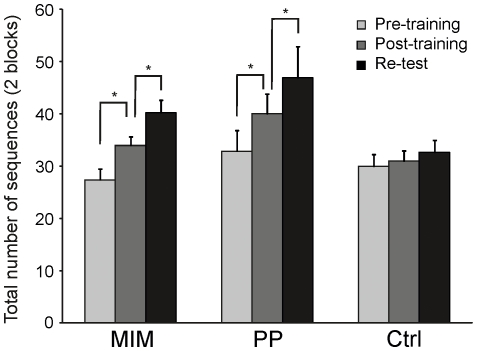
Effect of training on the total number of correct sequences. The total number of correct sequences performed during the two-block practice of the pre-training, the post-training and the re-test sessions, have been computed for the three different groups of subjects participating in Experiment #1. Both MIM and PP groups increased their performance significantly in the post-training session, while no significant difference was found in the Ctrl group. Similarly, during re-test, only in the MIM and PP groups, we found an increase in the total number of sequences, demonstrating the occurrence of an early boost effect. Error bars indicate one SD.

In the post-training session, the total number of correct sequences was 34.00±1.60, 40.00±3.85 and 31±1.92 in the MIM, PP and Ctrl groups, respectively. In the re-test session, these values further increased to 40.25±2.36 and 46.88±6.01 in the MIM and PP groups, respectively, but it remained stable in the Ctrl group (32.63±2.37) (Fig. 3). An ANOVA_RM_ on the number of correct sequences showed a main effect of session (F_2,42_ = 47.85, P<0.001) as well as a group×session interactions (F_4,42_ = 6.50, p<0.001), but no main effect of group (F_2,21_ = 2.13, p = 0.25). Post-hoc revealed that the MIM and PP groups significantly improved their performance between the pre- and post-training sessions (p<0.05 and p<0.01, respectively) and between the post-training and re-test sessions (p<0.05 and p<0.01, respectively). In contrast, subjects from the Ctrl group did not show any significant change in performance between the pre-training and post-training sessions (p = 0.62), and between the post-training and re-test sessions (p = 1) (see Fig. 3). These results indicate that, for this task, MIM training can lead to an early boost in performance, as previously demonstrated for PP training, while the exposure to the three practice sessions in the absence of a specific training, as in the Ctrl group, was insufficient to yield such a performance increase.

**Figure 4:**
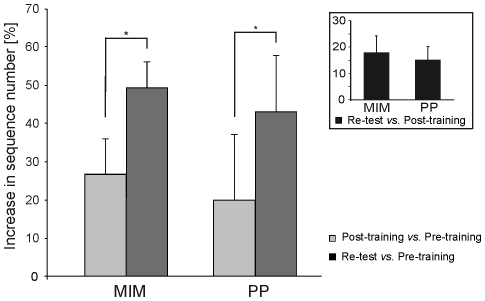
Early boost following MIM and PP training. The increase in the total number of sequences between the post-training and re-test sessions is expressed in percent with respect to data gathered in the pre-training session. This shows that MIM and PP training led to the same increase in performance in the post-training and re-tests sessions and confirms the occurrence of an early boost in both groups. The inset shows the amplitude of the early boost, estimated as the difference between the total number of sequences in the re-test and post-training sessions - in both groups and expressed in percent; no difference in early boost amplitude was found between the two groups. Error bars indicate one SD.

In the following analysis, we then focused on the relative increase in the total number of sequences in MIM and PP groups during the post-training and re-test sessions; because the previous analysis did not show any significant effect across sessions in the Ctrl group, it was excluded from these analyses. In the MIM and PP groups, the total number of sequences increased, respectively, by 27% and 20% between the pre- and post-training sessions, and by 49% and 43% between the pre- and re-test session (see Fig. 4). An ANOVA_RM_ on these relative increase in performance indicated a main effect of session (F_1,14_ = 9.25 p<0.01) but no effect of group (F_1,14_ = 0.78, p = 0.39) or a group×session interaction (F_1,14_ = 0.001, p = 0.97). Then, the magnitude of the early boost was quantified as the relative increase in the total number of sequences between the post-training and re-test sessions (see Methods). The early boost magnitude was 18% in the MIM group and 15% in the PP group (see inset of Fig. 4); these values were not statistically different across groups (one-way ANOVA, F_1,14_ = 0.46, p = 0.5). Altogether, these results indicate that the two types of training we investigated were equally efficient and that they both induced an early boost of comparable amplitude.

As far as the sequence duration is concerned, we found that, in the post-training session, it equaled 1.32±0.09 s in the MIM group, 1.16±0.13 s in the PP group and 1.40±0.13 s in the Ctrl group (mean ± SD, n = 8); it was 1.24±0.10 s, 1.08±0.11 s and 1.40±0.13 s in the re-test session, respectively. An ANOVA_RM_ showed a main effect of session (pre-training, post-training and re-test, F_2,42_ = 38.73, p<0.001) and a group×session interaction (F_4,*42*_ = 4.14, p<0.01) but no main effect of group (F_2,21_ = 1.47, p = 0.25). A post-hoc revealed that, in the MIM and PP groups, the mean duration of sequences decreased between the pre- and post-training sessions (p<0.001 and p<0.05, respectively), and continued to decrease in the re-test (all p<0.001). In contrast, in the Ctrl group, these values remained stable across sessions when compared to the pre-training session values (p = 0.62 in the post-test and p = 1 in the re-test).

In order to investigate further the training effect in the two experimental groups, we then focused on the relative change in sequence duration in the post-training and re-test sessions with respect to the pre-training session; as for the total number of sequences, data from the Ctrl group were not included in this analysis. The mean sequence duration decreased, respectively, by 22% and 16% between the pre- and post-training sessions in the MIM and PP groups and reached, for both groups, 27% in the retest session. An ANOVA_RM_ on these relative values did not revealed an effect of session (F_1,14_ = 4.05, p = 0.06) or an effect of group (F_1,14_ = 0.33, p = 0.57) or a group×session interaction (F_1,14_ = 0.65, p = 0.43).

During the training session, the mean sequence duration for the MIM group was 2.38±0.21 s and was significantly longer than in the pre-training session (paired *t*-test, t = −5.56, p<0.001). The rating of the vividness of mental images during MIM was 4.57±0.19. During the debriefing following MIM, all participants reported that they used the imagery type outlined in the scripts. They combined internal visual and kinaesthetic imagery without switching to external visual imagery. None reported changing the imagery script to suit their individual needs, and all rehearsed the motor sequence as they were requested to do it.

### Experiment #2: Role of M1 in the early boost in performance induced by MIM learning

As in Experiment #1, we first aimed to determine whether the M1 and Vertex groups were comparable in terms of performance during the pre-training session. The total number of sequences performed in the pre-training session was 26.75±3.52 for the Vertex group and 28.50±3.11 for the M1 group; their mean sequence duration was, respectively, 1.75±0.21 s and 1.65±0.12 s. Two separate one-way ANOVA on the total number of sequences (F_1,14_ = 0.13, p = 0.71) and on the mean sequence duration (F_1,14_ = 0.16, p = 0.69) showed no difference between groups.

**Figure 5:**
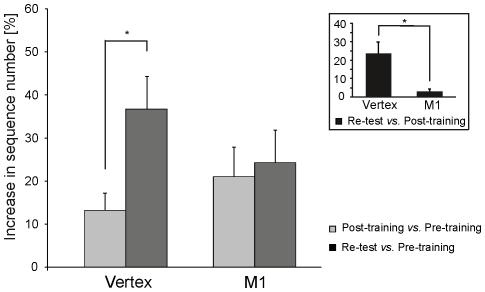
Lack of early boost following M1 cTBS. The increase in the total number of sequences in the post-training and re-test sessions is expressed in percent with respect to data gathered in the pre-training session. This shows that, whereas the performance in the Vertex and M1 groups was comparable in the post-training session, no early boost was found in the M1 group. This difference between groups was confirmed by data shown in the inset (same conventions as in Fig. 4). Error bars indicate one SD.

In the post-training session, the total number of sequences increased by 13±3.9% in the Vertex group and by 21±6.9% in the M1 group when compared with values from the pre-training session; in re-test session, this value reached 37%±7.6 in the Vertex group, but remained nearly stationary in the M1 group (24±7.5%) (Fig. 5). An ANOVA_RM_ on these relative values showed a main effect of session (F_1,14_ = 15.02, p<0.01) and a significant group×session interaction (F_1,14_ = 8.72, p<0.01), but no main effect of group (F_1,14_ = 0.08, p = 0.77). Post-hoc revealed no difference between the two groups when comparing the increase in the total number of sequences between the pre- and post-training sessions (p = 0.87), indicating that the outcome of the MIM training was comparable in both groups. However, a post-hoc revealed that the increase in the number of sequences between the post-training and re-test sessions was significant in the Vertex group (p<0.01), but not in the M1 group (p = 0.91). Indeed, the total number of sequences in the re-test session expressed in relative value with respect to the total number of sequences in the post-training session indicated that the magnitude of the early boost was 24% in the Vertex group but only 3% in the M1 group; this difference was statistically different (one-way ANOVA, F_1,14_ = 9.46, p<0.01) (see inset of Fig. 5). This finding clearly indicated that an inhibition of M1 as induced by cTBS and administered just after the MIM training session, impaired the early boost in performance.

The sequence duration was, in the post-training session, 1.44±0.12 s for the M1 group and 1.54±0.18 s for the Vertex group, which corresponded to a decrease of 13±3% and 11±3% for the two groups, respectively. These values further decreased to 1.35±0.10 s (18±3%) and 1.34±0.15 s (22±3%) in the re-test session in the M1 and Vertex group, respectively. An ANOVA_RM_ on these relative values showed a main effect of session (F_1,14_ = 24.91, p<0.001) but no main effect group (F_1,14_ = 0.17, p = 0.68) and no group×session interactions (F_1,14_ = 3.65, p = 0.07).

During the MIM training session, the mean sequence duration was 2.54 s (±0.17) in the M1 group and 2.78 s (±0.28) in the Vertex group. Again, we found that the duration of imagined sequences during the training session was significantly longer than that of the executed sequences during the pre-training session (paired *t*-test, t = −10.13, p<0.001). No group difference was found when comparing the self-reported individual ratings evaluating the vividness of the mental motor images, the mean scores were 4.50±0.19 in the M1 group and 4.48±0.18 in the Vertex group (one-way ANOVA, F_1,14_ = 0.22, p = 0.64). During the debriefing sessions following MIM, all participants reported that they used the type of imagery outlined in the instructions.

## Discussion

The first aim of the present study was to determine whether MIM training induces an early boost in performance as already reported following PP training [5], [11], [45]. We found that both MIM and PP trainings of a finger sequence yielded a significant, and comparable, increase in performance 30 minutes after learning ended, known as the “early boost”. Additionally, we aimed to investigate whether M1 plays a role in implementing this early boost and found that an inhibition of M1 performed immediately after MIM training altered the early boost, indicating that M1 is causally involved in this process, even if the movements to be learned were not executed during training.

Skill acquisition is typically accompanied by a significant increase in speed and accuracy. As expected, we found that participants improved their performance in a sequential finger-tapping task after PP training, but also after MIM training, as evaluated immediately in the post-training session; this finding is in accordance with results in the literature showing the efficacy of MIM training [“post]-[training” session,14,29,31,46–48]. Importantly, our control group involved in a reading task did not show any performance enhancement in the post-training session, indicating the specificity of this training effect. Furthermore, the present results show that, irrespective of the training procedure (MIM or PP), this initial increase in performance was followed by an early boost, namely a significant offline improvement occurring after a 30 minute resting period; the amplitude of this early boost was similar in both PP and MIM groups. Such a phenomenon has already been reported in the literature for PP training [5]–[8], [11], but, to the best of your knowledge, has never been described after MIM training. Following PP training, Hotermans and collaborators reported a 15% increase in performance in a similar finger sequence task after a 30 min rest period [5]. Our finding that an early boost, of comparable amplitude, also occurs after MIM training allows us to gain further insight into the behavioral analogy between MIM and PP [22], [23].

Another important finding of the present study is that an inhibition of the contralateral M1, as induced by cTBS applied over this area just after MIM training, prevented the occurrence of the early boost, while it remained unaltered in the Vertex group. This result is reminiscent of the finding of Hotermans et al. [11] who showed that interfering with M1 functioning by applying an off-line rTMS (1 Hz rTMS applied at 90% of the resting motor threshold during 20 min immediately after training) significantly reduced the early boost subsequent to a PP training. However, Hotermans et al. [11] reported that rTMS applied off-line over M1 only partly reduced the early boost amplitude by about 50%; indeed, they found that, following off-line rTMS, the early boost amplitude was only 8%, in comparison with the 15% found in the control condition. In contrast, the present study shows that the early boost following MIM training was nearly entirely abolished by cTBS (3% in the M1 group *vs.* 24% in the Vertex group). The interpretation of Hotermans et al. [11] for a partial decrease in the early boost after PP training was that the processes underlying the early boost in performance could be implemented in a more distributed manner, over a large cortico-subcortical network [13], [49] and therefore only partly altered when interfering with M1 functioning by means of rTMS. Although we used a different training procedure (MIM *vs.* PP), the present study rather suggests that a difference in the efficacy of rTMS *vs*. cTBS might explain this difference [38]. Alternatively, it is possible that the susceptibility of M1 to inhibition/interference is larger following MIM training because M1 activation has been shown to be less important during MIM when compared to PP [25]. Furthermore, using a finger sequence task, Lacourse et al. [24] reported that PP-related improvements during motor practice are associated primarily with an increased activation in the contralateral M1, whereas enhanced performance following MIM training was accompanied by a similar increase in contralateral M1, but to a lesser extent. The same conclusion about a comparable, but weaker, activation of M1 has been recently drawn for movement observation [9]. Based on these findings, we may hypothesize that, after MIM training or movement observation, M1 activation is weaker than following PP training, and could, therefore, be more susceptible to cTBS application.

Recently, Wohldmann et al. [50], [51] proposed that PP strengthens both an effector-dependent and independent representation of the task, whereas only the latter might be strengthened by MIM practice. In other words, motor representations activated during MIM could only concern the abstract representation of an effector-independent motor plan [52]. Interestingly, the existence of two functionally distinct subdivisions in M1 may account for this difference in M1 activation following MIM and PP training [53]–[55]. Indeed, based on differences in cytoarchitecture and neurotransmitter binding distributions, it has been shown that M1 is divided into a caudal (posterior area 4 or 4p) region and a rostral (anterior area 4 or 4a) region [53], [54]. Using a finger-thumb opposition sequence, Sharma et al. [55] showed that, while the activation of area 4p is somehow similar during MIM and PP, the activation of area 4a was significantly reduced during MIM when compared to PP. This suggests that area 4a may encode an effector-dependent representation of movements whereas area 4p would encode an effector-independent movement representation [55]. Therefore, as an alternative explanation for the nearly complete disappearance of the early boost following MIM training, it is sensible to assume that, because MIM training led mainly to the activation of one effector-independent representation in M1, this unique representation is more sensitive to cTBS than those activated by PP training.

Interestingly, following PP training, it has been shown that the early boost in performance is of short duration and disappears after 4–5 hours [5], [11]. Hotermans et al. [11] further reported that the early boost in performance observed 30 min after training is reduced by rTMS over M1 without having any detrimental consequences on the long-term behavioral improvement tested 4 h or 24 h after. This result suggests that M1 takes part in the behavioral enhancement observed during the early post-training period but does not play a critical role in long-lasting consolidation of motor skill, especially in the one taking place during sleep [56]. These findings are consistent with previous studies showing that rTMS over M1 can disrupt the consolidation process of an implicit motor sequence learning during daytime but not overnight [57]. Interestingly a recent study has suggested that M1 may also be involved in the so-called “reconsolidation” process, namely the extra changes that occur in an already consolidated motor memory [58]. This led these authors to suggest that M1 could serve as an “executive memory storage” involved in the interactions with the environment, and which would feed a “core memory storage” that may include, amongst others, the cerebellum and basal ganglia [56], [59]. Assessing the pattern of brain activations, and especially the involvement of M1 in the subsequent long-lasting phases of the consolidation process, such as during the reconsolidation of motor memory, should provide further insight into the neural correlates of MIM processes.

To conclude, our findings support the causal role of M1 in implementing the early boost of performance following MIM training on an explicit finger motor sequence. The present study also shows, for the first time, the emergence of an early boost in performance following MIM training, which further reveals some behavioral and neural similitude between MIM and PP [22], [23]. While the involvement of M1 in MIM has already been reported, the present results allow us to identify another critical role of M1, namely its contribution to the early boost process subsequent to MIM training. Such findings have strong theoretical and practical applications in both motor learning and motor rehabilitation, in which performing MIM is more cost-effective and easily feasible [18], [60]. Investigating in great detail motor memory consolidation processes of imagined movements could help us to ascertain how to schedule and perform MIM in order to develop effective training strategies.
